# Risk of myocardial infarction and stroke following microbiologically confirmed urinary tract infection: a self-controlled case series study using linked electronic health data

**DOI:** 10.1136/bmjopen-2024-097754

**Published:** 2025-06-30

**Authors:** Nicola F Reeve, Victoria Best, Rebecca Cannings-John, David Gillespie, Kathryn Hughes, Fiona V Lugg-Widger, Fatemeh Torabi, Mandy Wootton, Ashley Akbari, Haroon Ahmed

**Affiliations:** 1 Cardiff University, Cardiff, UK; 2 Swansea University, Swansea, UK; 3 Public Health Wales, Cardiff, UK

**Keywords:** Cardiology, Stroke, Urinary tract infections, Myocardial infarction

## Abstract

**Abstract:**

**Objectives:**

The inflammatory response from acute infection may trigger cardiovascular events. We aimed to estimate associations between microbiologically confirmed urinary tract infections (UTIs) and first acute myocardial infarction (MI) and stroke.

**Design:**

We used a self-controlled case series, with risk periods 1–7, 8–14, 15–28 and 29–90 days after UTI. Included individuals experienced the outcome and exposure of interest and acted as their own controls.

**Setting:**

We used individually linked general practice, hospital admission and microbiology data for the population of Wales held by the Secure Anonymised Information Linkage databank.

**Participants:**

Included individuals were Welsh residents aged over 30 years with a record of a hospital admission for MI or stroke (outcomes) and evidence of a microbiologically confirmed UTI (exposure) during the study period of 1 January 2010 to 31 December 2020.

**Main outcome measures:**

The primary outcome was acute MI or stroke identified using the International Classification of Disease V.10 codes from inpatient diagnoses recorded in the Patient Episode Database for Wales. We used Poisson regression to estimate incidence rate ratios (IRRs) and 95% CIs for MI and stroke during predefined risk periods, compared with baseline periods.

**Results:**

During the study period, 51 660 individuals had a hospital admission for MI, of whom 2320 (4.5%) had 3900 microbiologically confirmed UTIs, and 58 150 had a hospital admission for stroke, of whom 2840 (4.9%) had 4600 microbiologically confirmed UTIs. There were 120 MIs during risk periods and 2190 during baseline periods, with an increased risk of MI for 1–7 days following UTI (IRR 2.49, 95% CI (1.65 to 3.77)). There were 200 strokes during risk periods and 2640 during baseline periods, with an increased risk of stroke for 1–7 days following UTI (IRR 2.34, 95% CI (1.61 to 3.40)).

**Conclusions:**

UTI may be a trigger for MI or stroke. Further work is needed to understand mechanisms and test interventions to reduce the risk of cardiovascular events among people with UTIs in primary care.

**STRENGTHS AND LIMITATIONS OF THIS STUDY**

- The self-controlled case series methodology means that any time-invariant confounding is inherently controlled for, even if unmeasured.
- Multiple exposure definitions allowed the assessment of a wide spectrum of urinary tract infection presentations in line with clinical practice.
- The use of microbiology data in exposure ascertainment increases reliability compared with previous research.
- The use of routine data means that outcome ascertainment relies on accurate coding.
- Residual confounding may still be an issue.

## Introduction

 Myocardial infarction (MI) and stroke are leading causes of mortality.^1^ Alongside well-established modifiable risk factors such as hypertension, low-density lipoprotein cholesterol and tobacco smoke,^2^ there is increasing interest in the role of acute infection in the pathogenesis of cardiovascular disease.^3^ Excess mortality from cardiovascular disease during influenza epidemics was first reported in the 1930s,^4^ but more robust associations between acute infections and acute cardiovascular events were only appreciated over the past few decades.^3^ Three main mechanisms have been proposed. First, the inflammatory response from acute infection may destabilise atherosclerotic plaques. Second, the prothrombotic, procoagulant state associated with acute infection may increase the risk of thrombosis at the site of plaque disruption. Third, tachycardia driven by inflammation and fever may cause ‘demand ischemia’ if the metabolic demands of the myocardial cells exceed oxygen supply.^3^

Most associations between acute infection and cardiovascular disease arose from observational studies of acute respiratory tract infections and acute MI.^35^,^11^ The risk of MI increases during and after a respiratory tract infection, and the magnitude varies according to organism.^8^ Increased short- and long-term risk of cardiovascular events has also been reported for pneumonia, sepsis, bacteraemia and COVID-19.^12^,^21^ However, associations with other infections, particularly those with bacterial aetiology, have been less well studied. Urinary tract infections (UTIs) are one of the most common bacterial infections seen in primary care,^22^ with 37% of women reporting having had at least one UTI in their lifetime and 29% experiencing more than one.^23^ Pathogens in the urinary tract are recognised by the innate immune system and induce a rapid and robust pro-inflammatory response,^24^ which could plausibly trigger a cardiovascular event by any of the three mechanisms described above. To our knowledge, only one study has examined the association between UTIs and acute MI and stroke.^9^ This study used electronic health record data from the General Practice Research Database and ascertained UTI from clinical codes reported by general practitioners in UK general practice. This raises questions about diagnostic certainty and misclassification, especially given data from multicountry cohort studies showing that roughly two-thirds of women with UTI suspected in primary care had no evidence of bacteria on microbiological culture.^25^

Determining associations between UTI and MI or stroke has potentially important clinical implications and could justify subsequent clinical trials of preventative therapy, such as antiplatelet drugs prescribed alongside antibiotic treatment. Therefore, this study aimed to estimate associations between microbiologically confirmed UTIs and MI or stroke using a self-controlled case series (SCCS) design and linked population-scale primary care and microbiology data.

## Methods

### Study design and population

Study procedures are described in detail in the published study protocol.^26^ We used an SCCS design where individuals act as their own controls, inherently controlling for time-invariant within-subject confounders, even when these are unmeasured or unknown.^27 28^ The SCCS design starts with individuals who have experienced an outcome of interest and aims to answer the question ‘when did this outcome occur?’ rather than ‘how often did this outcome occur?’. The incidence of each outcome is calculated for prespecified risk periods and compared with baseline (control) periods. The unit of measurement is person-days. The model assumptions, how they apply to our study, and the solutions to violations of the assumptions are given in online supplemental e-table 1.

For this SCCS, the source population were individuals with good quality data linkage within the Secure Anonymised Information Linkage (SAIL) Databank, an ISO27001 certified trusted research environment (TRE) for anonymised individual-level population-scale data.^29^ Good quality linkage is defined as individuals for whom either (1) NHS number passes check digit test, (2) surname, first name, postcode, date of birth and gender code match exactly to Welsh Demographic Service or (3) fuzzy matching probability ≥0.9. We included individuals who were Welsh residents, aged 30–100 years, with a hospital admission record for MI or stroke (recorded as a primary or secondary diagnosis in hospital data). We included only the first acute MI or stroke, occurring during the observation period of 1 January 2010 to 31 December 2020. We chose a lower age bound of 30 years to reduce the chance of including MIs and strokes due to congenital or other non-atherosclerotic causes.

### Data sources

We used the SAIL Databank to access the following linked data: Welsh Longitudinal General Practice (WLGP) data, Patient Episode Database for Wales (PEDW) and the Welsh Results Reporting Service (WRRS). The WLGP data contain anonymised individual-level data from people registered with 86% of general practices in Wales, equating to longitudinal data for 2∙6 million people.^30^ It includes demographic data, acute and chronic clinical diagnoses and prescription data. The PEDW data contain International Classification of Disease Version 10 (ICD-10)-coded diagnoses for individuals admitted to any Welsh hospital and Welsh residents treated in English hospitals.^31^ The WRRS data include all tests requested from primary and secondary care NHS Wales organisations processed and analysed in NHS Wales laboratories, including requests for urine microscopy and culture.^32^ This data is generated using the same Standard Operating Procedure, as all Welsh microbiology laboratories are part of the same network. Data availability across these data sources varies according to when clinical information systems began, with data quality and completeness improving over time (see online metadata or online supplemental e-table 2 for more details).^30^,^32^

### Exposures and outcomes

The exposure of interest was incident UTIs. To be defined as ‘incident’, a UTI required a 7-day period between the earliest date of that episode and the latest date of a previous episode. UTIs within 7 days of each other were grouped as single episodes, with a hierarchical approach used to assign the date of exposure and determine which analysis that episode would contribute to (see online supplemental e-figure 1). To ascertain microbiologically confirmed UTIs, we developed definitions that reflected the Public Health Wales Microbiology Division’s standard operating procedure for urine investigation.^33^ The standard operating procedure is based on the Standards for Microbiology Investigations developed by the UK Health Security Agency.^34^ These procedures are followed by NHS microbiology laboratories across Wales. For each definition, the data sources required and the clinical scenario represented are summarised in table 1, and relevant code lists are provided in online supplemental tables 3–5.

**Table 1:** Definitions of urinary tract infection for primary and secondary analyses

	Urine culture results in WRRS	Time frame	Clinical scenario
Primary analysis	Yes, showing bacterial growth of ≥10^8^ cfu/L and WBC ≥10^8^ /L	7-day window	GP clinically suspected and microbiologically confirmed UTI
Secondary analysis 1	Yes, showing mixed bacterial growth (any descriptor for ‘mixed growth’ or >3 organisms).	7-day window	GP clinically suspected UTI with mixed growth
Secondary analysis 2	No	Same day	GP clinically diagnosed and treated UTI. It is important to consider this group, as not all individuals with suspected UTIs have urine cultures, and limiting to those with cultures is subject to selection bias.
Secondary analysis 3	Yes, showing bacterial growth of <10^7^ cfu/L	7-day window	UTI is clinically suspected but not supported by microbiology. This group is important to understand if early symptoms and signs of acute MI or stroke are attributed to UTI.

As the urinary tract infection definitions are combinations of two or more components, the start of the risk period is defined as the date of the earliest component. The components are required to occur within the given timeframe. All analyses include the following components: UTI-related diagnostic or symptom Read code in GP data (online supplemental e-table 3); Antibiotic prescription in GP data (online supplemental e-table 4). Some analyses include a third component, urine culture results in WRRS, as indicated above.

cfu, colony forming units ; GP, general practitioner; MI, myocardial infarction; UTI, urinary tract infection; WBC, white blood cell; WRRS, Welsh Results Reporting Service.

In our primary analysis, an individual was regarded as being exposed to a UTI if all of the following events occurred within a 7-day window:

1. A GP record of a UTI diagnostic or symptom Read code (WLGP data).
2. A GP record of an antibiotic prescription concordant with UTI treatment (WLGP data).
3. A microbiology record of a urine sample with bacterial growth of a single organism of ≥10^8^ colony forming units (cfu) per litre and white blood cells ≥10^8^/L (WRRS data). If two organisms were grown, both needed to demonstrate growth of ≥10^8^ cfu per litre. More than two organisms were regarded as mixed growth and thus not supportive of a microbiologically confirmed UTI.

The primary analysis represents the clinical scenario of a GP clinically suspected and microbiologically confirmed UTI. For all analyses, the date of a UTI was defined as the earliest date of occurrence of any of the events necessary for each different UTI definition. Therefore, in the primary analysis, the UTI date was the earliest of the following events: UTI-related diagnostic code, antibiotic prescription and supporting urine culture specimen collection date.

We undertook secondary analyses where we estimated the risk of MI and stroke among individuals with a clinically suspected UTI with mixed growth on culture. This analysis was included to address the uncertain clinical significance of mixed bacterial growth in an individual with symptoms of UTI. We also estimated the risk of MI and stroke among individuals with a clinically suspected and treated UTI (no microbiology) and among individuals where UTI was clinically suspected and treated but not supported by microbiology (no bacterial growth) (table 1).

Outcomes of interest were acute MI or stroke identified using ICD-10 codes (online supplemental e-table 5) from inpatient diagnoses recorded in PEDW. We only included the first acute MI or stroke diagnosis in the observation period. Individuals meeting the primary analysis criteria for UTI exposure were selected from cases of MI or stroke.

### Risk periods

**Figure 1:**
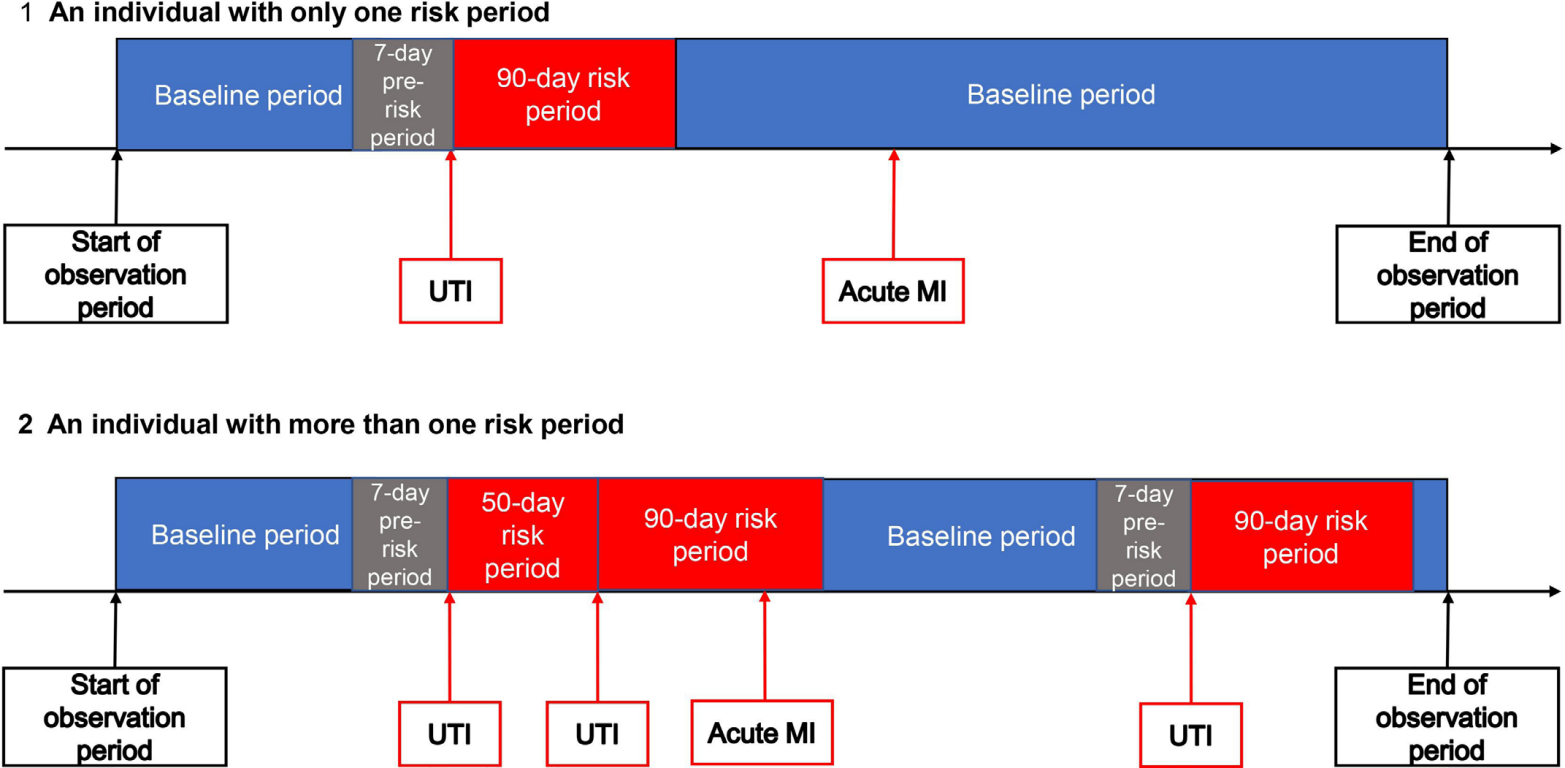
Diagrammatic representation of observation time for an individual in the proposed self-controlled case series design. UTI, urinary tract infection.

A diagrammatic representation of observation time for individuals in the SCCS is given in figure 1. Based on previous research,^9^ our predefined risk period (risk of MI or stroke following a UTI) was 0–90 days, with day 0 being the date of the UTI (figure 1, scenario 1). Individuals could have more than one UTI during the observation period and, therefore, more than one 90-day risk period. Where risk periods overlapped (figure 1, scenario 2), the later period took precedence and the earlier period was shortened. We included a prerisk period of 7 days before the UTI diagnosis to allow for the situation where an individual had a UTI for several days prior to consultation. This ensured that MIs or strokes in this period were not erroneously attributed to the baseline period. Baseline periods were all other times aside from risk and prerisk periods.

### Statistical analysis

We described the cohort of individuals aged 30–100 years who were resident in Wales and experienced an MI or stroke between 1 January 2010 and 31 December 2020 with respect to age, sex, history of diagnoses and prescription drugs prior to their MI or stroke, smoking status, Welsh Index of Multiple Deprivation V.2019^35^ and electronic Frailty Index. Characteristics were ascertained from WLGP data, with the cohort having data available for a median of 13 years (25th to 75th centiles 8–16 years) prior to cohort entry. From this cohort of individuals with MI or stroke, those who also experienced at least one UTI were included in the SCCS.

We used conditional logistic regression to estimate incidence rate ratios (IRRs), with 95% CIs, for the risk of acute MI or stroke in prerisk and risk periods compared with baseline periods. The SCCS design inherently controls for time-invariant covariates such as sex. We adjusted each model for age, season and year of UTI diagnosis. Age and season are associated with the incidence of UTI, MI and stroke. The year of UTI diagnosis was included because diagnostic and coding practices may have changed over time as a result of guidance and antibiotic stewardship policies. We reported crude and adjusted IRRs for the risk of MI or stroke in the prerisk period and the risk period (at intervals of 0, 1–7, 8–14, 15–28 and 29–90 days after UTI), compared with baseline periods.

We undertook several predefined sensitivity analyses to assess the robustness of our findings:

1. We explored the impact of using a wider definition of MI and stroke, including ICD-10 codes for acute coronary syndromes and transient ischaemic attacks.
2. We explored the impact of widening the microbiological definition of UTI to bacterial growth of a single organism of ≥10^7^ cfu per litre irrespective of white blood cell count.
3. We differentiated first-ever MI or stroke from recurrent events. This analysis excludes individuals with a PEDW record of an event before the observation period and includes only those who have their first-ever event during the observation period.
4. We extended the prerisk period to 14 days.
5. We repeated the analysis excluding individuals who died within 30 days of an event to examine the potential effect of an event-dependent observation period.
6. We restricted the definition of UTI to include only antibiotic prescriptions for nitrofurantoin (currently recommended first-line therapy in the UK) to explore whether the choice of antibiotic impacted the relationship. Separately, we included only antibiotic prescriptions for trimethoprim.
7. We examined whether the COVID-19 pandemic may have affected our findings by (1) excluding individuals whose MI or stroke occurred in 2020 and ending the observation period at the end of 2019 and (2) including an interaction term to explore whether the association between exposure and outcome differed in 2020 versus pre-2020.

We also explored differential effects in subgroups in the primary analysis based on bacterial organism (*Escherichia coli (E. coli)* vs other organisms) and history of diabetes (given its potential role as a risk factor for both UTI and MI/stroke).^36 37^

All study data were held within the SAIL Databank. Data access, research permissions and approvals were obtained from the SAIL independent Information Governance Review Panel (IGRP), project number 0972. Analyses were conducted within the SAIL TRE with strict disclosure control processes in place. Only aggregated outputs were approved for release to ensure individuals were not identified (all counts in this paper are rounded to the nearest 10; counts less than five were suppressed and denoted as such). Analyses were undertaken in R V.4.1.3, using the SCCS package V.1.5.

### Patient and public involvement

We developed this research in collaboration with members of the Wales Centre for Primary and Emergency Care Research Service Users group (SUPER) and the SAIL consumer panel. We consulted SUPER and the SAIL consumer panel regarding all stages of this research, including discussion of analysis plans, review of findings and plans for dissemination (eg, public facing outputs). We extended our subgroup analysis to include individuals with diabetes in response to discussions with the SAIL consumer panel members.

## Results

**Figure 2:**
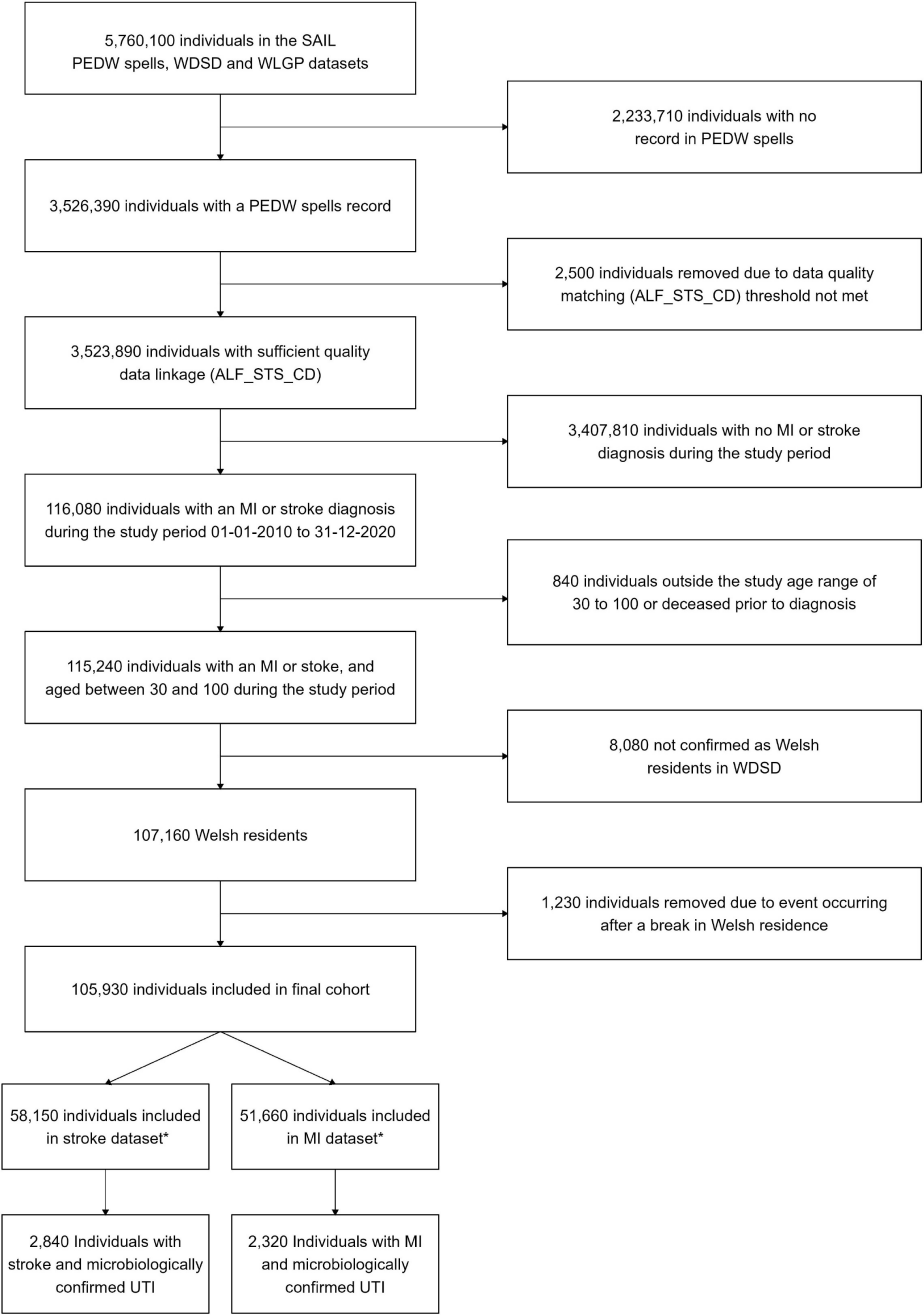
Selection of myocardial infarction (MI) and stroke cases. *Individuals appear and are counted in both the stroke and MI datasets if they had both an MI and a stroke event within the study period. All counts are rounded to the nearest 10. PEDW, Patient Episode Database for Wales; UTI, urinary tract infection; WDSD, Welsh Demorgraphic Service Dataset WLGP, Welsh Longitudinal General Practice.

We identified 51 660 individuals with an ICD-10 code for MI and 58 150 with an ICD-10 code for stroke (figure 2). MI cases were 63% male. Median age was 77 years (25th to 75th centiles 66–85 years) for females and 69 years (25th to 75th centiles 59–78 years) for males. Stroke cases were 49% male, with median ages of 79 years (25th to 75th centiles 69–87 years) for females and 74 years (25th to 75th centiles 64–82 years) for males. Of these 51 660 individuals with MI and 58 150 with stroke, 2320 and 2840, respectively, also had at least one clinically suspected and microbiologically confirmed UTI during their observation period and were included in the primary analysis. The number of individuals and the number of exposures to UTI included in each analysis are given in online supplemental e-table 6. 1560 (67%) individuals with MI and 1990 (70%) with stroke had just one UTI during the observation period. 410 (18%) with MI and 460 (16%) with stroke had two UTIs, and 50 (2%) with MI and 70 (2%) with stroke had more than five UTIs (online supplemental e-table 7). The number of UTIs prescribed each antibiotic in the primary analysis is given in online supplemental e-table 8. A description of the characteristics of individuals experiencing both UTI and either MI or stroke is given in table 2. All data were complete, except ethnicity data. 68% of stroke cases and 55% of MI cases had no ethnicity recorded.

**Table 2:** Characteristics of cases of stroke and myocardial infarction, identified with a urinary tract infection

	Stroke*	MI*
Total number of cases	2840	2320
Sex (n, % male)	900 (31.5%)	900 (38.8%)
Age of males, years (median (25th, 75th centiles))	78 (70, 84)	74 (66, 81)
Age of females, years (median (25th, 75th centiles))	79 (71, 85)	76 (68, 83)
Ethnicity		
White (n, % non-missing)	900 (99.4%)	750 (98.6%)
Non-white (n, % non-missing)	10 (0.6%)	10 (1.4%)
Missing (n, %)	1940 (68.1%)	1560 (54.7%)
Welsh Index of Multiple Deprivation (WIMD) 2019 quintiles†		
1 (most deprived) (n, %)	480 (16.9%)	440 (18.8%)
2	550 (19.2%)	470 (20.1%)
3	620 (21.7%)	480 (20.8%)
4	600 (21.1%)	480 (20.9%)
5 (least deprived)	600 (21.1%)	450 (19.4%)
Current smoker (n, %)	410 (14.5%)	440 (18.9%)
Electronic frailty index (eFI) (mean (SD))	0.24 (0.11)	0.23 (0.11)
Prescribed lipid lowering drugs (n, %)	1870 (65.8%)	1580 (68.1%)
Prescribed aspirin (n, %)	1620 (57.1%)	1330 (57.6%)
Prescribed hypertensive drugs (n, %)	2200 (77.3%)	1760 (75.8%)
Prescribed beta blockers (n, %)	1510 (53.2%)	1220 (52.6%)
Chronic kidney disease (n, %)	830 (29.3%)	680 (29.5%)
COPD (n, %)	340 (11.8%)	320 (13.9%)
Asthma (n, %)	560 (19.7%)	490 (21.0%)
Hypertension (n, %)	1910 (67.2%)	1500 (64.6%)
Diabetes (n, %)	840 (29.6%)	710 (30.6%)
Cardiovascular disease (n, %)	2080 (73.0%)	1670 (72.1%)
Coronary heart disease (n, %)	610 (21.4%)	660 (28.4%)
Atrial fibrillation (n, %)	580 (20.6%)	290 (12.4%)
Heart failure (n, %)	300 (10.7%)	270 (11.6%)
Peripheral vascular disease (n, %)	270 (9.5%)	250 (10.7%)
Angina (n, %)	420 (14.9%)	520 (22.5%)
Transient ischaemic attacks (n, %)	380 (13.4%)	160 (7.1%)

* Counts rounded to the nearest 10.

† LSOA V.2011 and WIMD V.2019.

MI, myocardial infarction.

### Primary analysis

#### Myocardial infarction

**Figure 3:**
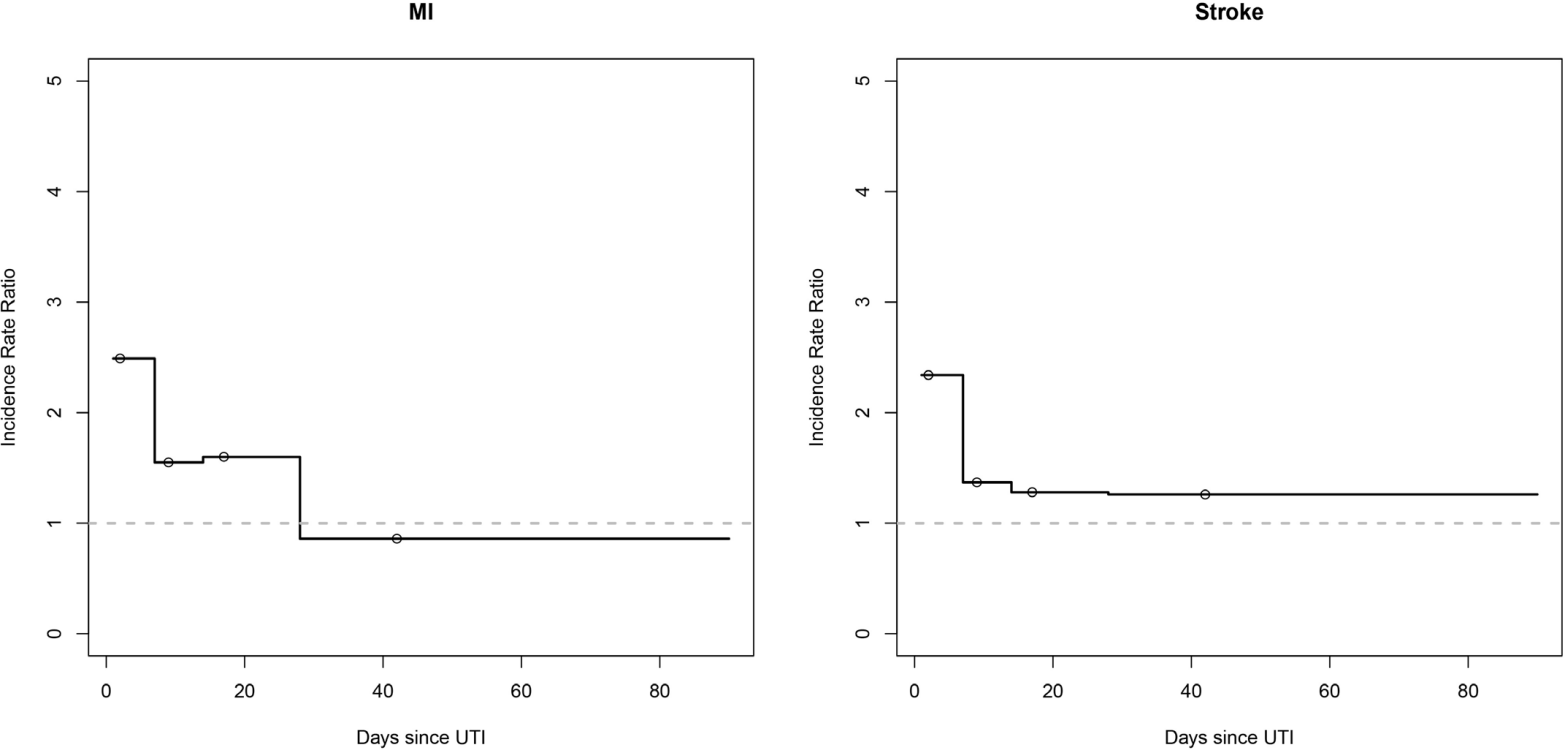
Incidence rate ratios for myocardial infarction and stroke after urinary tract infection from primary analysis. UTI, urinary tract infection.

In the primary analysis, 2320 individuals with MI had 3900 microbiologically confirmed UTIs. Fewer than five MIs occurred in the prerisk period, 120 MIs occurred in the risk period and 2190 occurred during baseline periods. Total observation time was 27 300 days for the prerisk period, 315 530 days for the risk period and 8 102 743 days for baseline periods. The risk of MI increased in the first 7 days following UTI (adjusted IRR 2.49, 95% CI (1.65 to 3.77)). There was no statistically significant increase in risk 8–14 days after UTI, but a further period of increased risk during 15–28 days after UTI (adjusted IRR 1.60, 95% CI 1.10–2.33) (figure 3 and table 3).

**Table 3:** Crude and age-, season- and year-adjusted incidence rate ratio (IRR) for first myocardial infarction (n=2320) and first stroke (n=2840) in periods after urinary tract infection compared with baseline time

Time period	MI				Stroke			
	No events*	Total Obs time (days)	Crude IRR (95% CI)	Adjusted IRR (95% CI)	No events*	Total Obs time (days)	Crude IRR (95% CI)	Adjusted IRR (95% CI)
Baseline	2190	8 102 743	1	1	2640	9 620 009	1	1
Prerisk	≤5	27 300†	‡	0.32 (0.10 to 1.00)	10	32 179	0.78 (0.37 to 1.63)	0.58 (0.28 to 1.23)
1–7 days post UTI	20	27 049	3.09 (2.04 to 4.66)	2.49 (1.65 to 3.77)	30	31 944	3.13 (2.15 to 4.55)	2.34 (1.61 to 3.40)
8–14 days	10	26 393	1.93 (1.14 to 3.26)	1.55 (0.92 to 2.63)	20	31 196	1.83 (1.12 to 3.00)	1.37 (0.84 to 2.24)
15–28 days	30	51 091	1.99 (1.37 to 2.90)	1.60 (1.10 to 2.33)	30	60 373	1.72 (1.19 to 2.48)	1.28 (0.88 to 1.85)
29–90 days	60	207 099	1.08 (0.83 to 1.39)	0.86 (0.67 to 1.12)	120	246 829	1.72 (1.43 to 2.07)	1.26 (1.05 to 1.52)

* Rounded to the nearest 10.

† Excluded for disclosure reasons.

‡ Rounded to the nearest 100.

IRR, incidence rate ratio; MI, myocardial infarction; UTI, urinary tract infection.

### Stroke

For stroke, 2840 individuals had 4600 microbiologically confirmed UTIs. 10 strokes occurred in the prerisk period, 200 in the risk period and 2640 during baseline periods. Total observation time was 32 179 days for the prerisk period, 374 939 days for the risk period and 9 620 009 days for baseline periods. The risk of stroke increased in the first 7 days following UTI (adjusted IRR 2.34, 95% CI (1.61 to 3.40)). There was no statistically significant increase in risk 8–28 days after UTI, but a further period of increased risk during 29–90 days after UTI (adjusted IRR 1.26, 95% CI 1.05 to 1.52) (figure 3 and table 3).

### Secondary analyses

**Figure 4:**
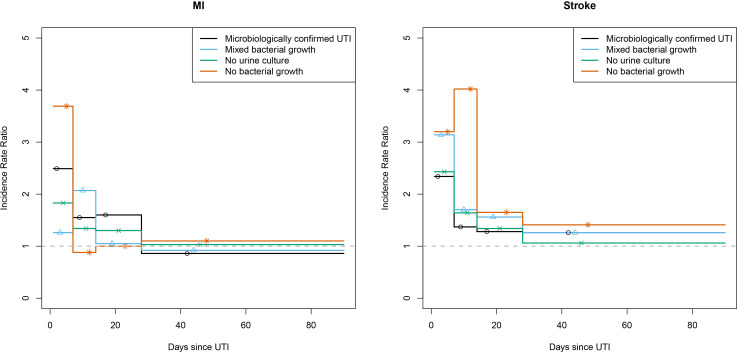
Incidence rate ratios for myocardial infarction after urinary tract infection (UTI) from primary and secondary analyses.

Among individuals with a clinically suspected UTI with mixed bacterial growth on culture, the adjusted IRR for MI in the first 7 days following UTI was 1.26 (95% CI (0.52 to 3.05)) and for 8–14 days after UTI was 2.07 (95% CI 1.03–4.15). Among individuals with clinically suspected UTI where there was no urine culture, the risk of MI was greater in the first 7 days after consultation (IRR 1.83, 95% CI (1.54 to 2.18)). The risk of MI was also elevated among those with clinically suspected UTI with no bacterial growth on culture (IRR 3.69, 95% CI (2.28 to 5.96)) (figure 4 and online supplemental e-tables 9–11). The risk of stroke was greater in the 7 days after UTI in all secondary analyses (figure 4 and online supplemental e-tables 9–11).

### Subgroup analyses

The interaction between UTI exposure and infection with *E. coli* was statistically significant (p value <0.0001 for both MI and stroke). The effect of infection with *E. coli* compared with other organisms was not consistent for MI and stroke: the magnitude of risk for MI was smaller for *E. coli* (IRR 2.55 (95% CI 1.37 to 4.76) for *E. coli*, 3.54 (95% CI 1.58 to 7.91) for other organisms), but the risk of stroke was higher for *E. coli* (IRR 3.06 (95% CI 1.87 to 5.00) for *E. coli*, 2.34 (95% CI 1.05 to 5.23) for other organisms). The interaction between UTI exposure and diabetes status was not statistically significant for either MI (p value 0.329) or stroke (p value 0.511) (online supplemental e-table 12).

### Sensitivity analyses

The results remained robust to the alterations of assumptions made in our sensitivity analyses. Adjusted IRRs were lower than crude IRRs for all analyses, with age being the main factor driving the attenuation of risk. Full results of the secondary and sensitivity analyses are given in online supplemental material.

## Discussion

### Main finding

In this SCCS, we found that microbiologically confirmed UTIs were associated with an increased risk of MI and stroke. The risk of MI was highest in the first 7 days after UTI, with a further period of increased risk 15–28 days after UTI. The risk of stroke was also highest in the first 7 days after UTI and was raised 29–90 days after UTI. In analyses including individuals with no urine culture, mixed growth on culture and no growth on culture, we found an increased risk of MI and stroke following clinically suspected UTI, though the mixed growth group had a delay before the observed increase in risk of MI. The group with no growth on culture had a marked increase in risk of both MI and stroke.

### Strengths and limitations

We used microbiology data to increase the reliability of exposure ascertainment. This is a key strength of this study and reduces the risk of misclassification in the primary analysis. It has also allowed an exploration of the implications of different microbiology definitions. To our knowledge, ours is the only study which uses microbiologically confirmed UTI to study the risk of MI or stroke following UTI. All microbiology laboratories across Wales use standardised procedures, meaning microbiological data are comparable across laboratories. We used the SCCS methodology, which meant that unmeasured time-invariant confounding from important differences between individuals who do and do not experience UTIs was eliminated. Measured and unmeasured characteristics that vary between individuals were inherently controlled. We also controlled for the effects of age, season and year. However, the findings could still be affected by residual confounding, such as from measurement error in adjusted covariates. A potential weakness of our approach is that we do not know the precise date of the onset of UTI, only the date of diagnosis. However, individuals are unlikely to be experiencing symptoms for more than a few days before seeking medical care,^25^ and this time period is included in the prerisk period to prevent erroneous attribution of MI or stroke during this time to baseline periods. We had no information on UTIs for which people did not seek medical care. We may also have unrecorded UTIs in WRRS prior to 2015 due to differences in recording practices. Any cardiovascular event subsequent to an unrecorded UTI would be assigned to baseline time, biasing our estimates towards the null.

The use of routine data means that results can be sensitive to the precise list of codes used for definitions of MI, stroke and UTI. We conducted a range of sensitivity analyses to examine this and found our results were robust to different definitions and to the choice of antibiotic. Secondary analyses are exploratory, and there is uncertainty in estimates due to small numbers of events, but an increase in risk of MI or stroke after UTI is still observed. The effect estimate for stroke did not return to baseline within 90 days, but the magnitude of incidence rates after 28 days is small, and even after taking this elevation into account, the increased rates seen immediately after UTI are significantly raised. It is possible that individuals were experiencing another source of inflammation at the same time as UTI, which could have contributed to the risk of MI or stroke. However, we believe the number of individuals affected by this to be relatively small.

### Comparison to the literature

The link between respiratory tract infections and MI or stroke has been well established.^5^,^1116^ Studies of influenza, respiratory syncytial virus, pneumonia and other respiratory viruses have found an increased risk of acute MI in the 1–3 days following a respiratory tract infection, with the effect sizes ranging from a threefold to sixfold increase according to the infecting organism.^5^,^11^ Several studies have also observed an association between pneumonia and acute cardiovascular events (including MI and stroke).^16^,^1938^ The link with UTI has been less well studied. Smeeth *et al* estimated an IRR of 1.66 for MI and 2.72 for stroke in the 1–3 days after UTI.^9^ Our results are in broad agreement with this: our secondary analysis with no urine culture has a similar definition to Smeeth *et al* and found an IRR of 1.83 for MI and 2.43 for stroke in the 1–7 days after UTI. While the effect sizes are smaller than those typically found for respiratory tract infections, incidence rates infer substantially increased risk post-UTI compared with baseline periods.

### Implications and future work

The mechanism behind the increased risk of MI and stroke includes destabilisation of atherosclerotic plaques by the inflammatory response, an increased risk of thrombosis and an increased heart rate contributing to demand ischaemia.^3^ Future work into how these mechanisms impact each of the groups of individuals examined in this study (microbiologically confirmed UTI; mixed growth on culture; no microbiology and no growth on culture) would provide a valuable aid to understanding, and preventing, the increased risk of cardiovascular events. The mechanism behind the increased risk is especially unclear for individuals with no growth on culture, where UTI is clinically suspected, but microbiology does not support the diagnosis and warrants further investigation. The increased risk in this group could be due to false negative culture results, perhaps due to atypical pathogens, low counts of bacteria or cultures taken after antibiotic treatment was started. It could be that early signs of MI or stroke are misdiagnosed as UTI, or it could be that it is the inflammatory response from the illness, which presents like an infection syndrome, that is important, rather than the specific infection itself. A better understanding of the mechanisms could lead to preventative treatment for at-risk individuals when diagnosed with UTI and fewer cardiovascular events. Recent work has set a precedent for this; the NIHR HTA-funded ASPECT trial is currently randomising people hospitalised with pneumonia to aspirin versus placebo to assess the effect on major adverse cardiovascular events.^39^

### Conclusions

We observed an increased risk of MI and stroke immediately following a UTI. This finding was robust to a range of secondary and sensitivity analyses and warrants further work to better understand mechanisms and inform trials of primary prevention.

## Supplementary material

10.1136/bmjopen-2024-097754 online supplemental file 1

## Data Availability

Data may be obtained from a third party and are not publicly available.
