# Supplementary material for: Risk of myocardial infarction and stroke following microbiologically confirmed urinary tract infection: a self-controlled case series study using linked electronic health data
Source: BMJ Open. 2025 Jun 30;15(6):e097754. doi: 10.1136/bmjopen-2024-097754 (PMC12211855; doi:10.1136/bmjopen-2024-097754)
Supplement: online supplemental file 1 [file bmjopen-15-6-s001.docx]

**Myocardial Infarction and stroke subsequent to urinary tract infection (MISSOURI): a self-controlled case series using linked electronic health records: Supplementary Material**

**Contents**

[e-Figure 1 Hierarchy applied to UTI episodes, to establish exposure date and which analysis the episode will contribute to 2](#_Toc159587972)

[e-Table 1 Model assumptions and solutions to violations of those assumptions 3](#_Toc188975690)

[e-Table 2 Description of SAIL datasets and the coverage of the Welsh population 4](#_Toc188975691)

[e-Table 3 Read codes for urinary tract infection to determine risk periods 5](#_Toc188975692)

[e-Table 4 Read codes for antibiotics to determine risk periods 9](#_Toc188975693)

[e-Table 5 ICD-10 codes for acute myocardial infarction and stroke to identify potential cases 10](#_Toc188975694)

[e-Table 6 Number of individuals and exposures to urinary tract infection included in each analysis. MI= Myocardial Infarction. 11](#_Toc188975695)

[e-Table 7 Number of UTIs per individual in the primary analysis. MI=Myocardial Infarction. 11](#_Toc188975696)

[e-Table 8 Number of urinary tract infections prescribed each antibiotic in the primary analysis. MI= Myocardial Infarction. 11](#_Toc188975697)

[e-Table 9 Crude, and age-, season- and year-adjusted incidence rate ratio (IRR) for myocardial infarction and stroke in periods after urinary tract infection compared with baseline time for secondary analysis 1: mixed bacterial growth on culture. MI= Myocardial Infarction. IRR= Incidence Rate Ratio. CI= Confidence Interval. 12](#_Toc188975698)

[e-Table 10 Crude, and age-, season- and year-adjusted incidence rate ratio (IRR) for myocardial infarction and stroke in periods after urinary tract infection compared with baseline time for secondary analysis 2: clinically diagnosed and treated urinary tract infection. MI= Myocardial Infarction. IRR= Incidence Rate Ratio. CI=Confidence Interval. 13](#_Toc188975699)

[e-Table 11 Crude, and age-, season- and year-adjusted incidence rate ratio (IRR) for myocardial infarction and stroke in periods after urinary tract infection compared with baseline time for secondary analysis 3: no growth on culture. MI= Myocardial Infarction. IRR= Incidence Rate Ratio. CI= Confidence Interval. 13](#_Toc188975700)

[e-Table 12 Age-, season- and year-adjusted incidence rate ratio (IRR) for myocardial infarction and stroke in the first seven days after urinary tract infection compared with baseline time for subgroup and sensitivity analyses. Subsequent risk periods are excluded for brevity. MI= Myocardial Infarction. IRR= Incidence Rate Ratio. CI= Confidence Interval 14](#_Toc188975701)

**
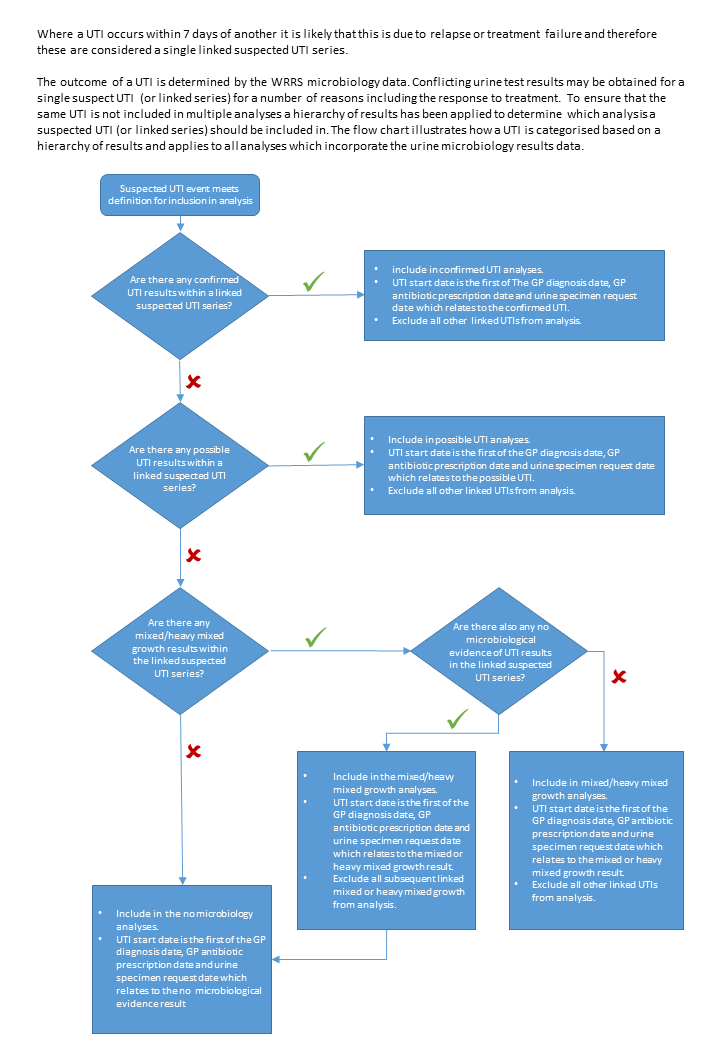
**

e-Figure 1 Hierarchy applied to UTI episodes, to establish exposure date and which analysis the episode will contribute to

**The SCCS method makes several assumptions that need to be met in order to obtain valid and unbiased estimates. In practice, these assumptions are not always met. There are model extensions which provide solutions to violations of these assumptions under certain circumstances.[34] The model assumptions, how they apply to our study, and the solutions to violations of the assumptions are given in e-Table 1.**

| **Assumption** | **How the assumption applies to this study** | **Solution** | **Example of use of the solution in the literature** |
| --- | --- | --- | --- |
| **Subsequent exposures should not be affected by previous events.** | **We might see a temporary increase in UTIs subsequent to an MI or stroke event, which would bias estimates towards the null.** | **Apply a pre-risk period.** | **Gibson et al. studied the association between prescription drugs and road traffic accidents. As some drugs may be used to treat anxiety or pain caused by the crash, a 4-week pre-exposure period was included.**(1) |
|  | **As both MI and stroke have relatively high death rates, the length of the observation period is dependent on events, and no further exposures are possible after death.** | **Conduct a sensitivity analysis that repeats the analysis, excluding individuals who died within 30 days of the event.** | **Langan et al. studied the risk of stroke following herpes zoster. They conducted a sensitivity analysis excluding individuals who died within 90 days of stroke.**(2) |
| **Event rates are constant within defined periods** | **MI and stroke are more common in older individuals and may be affected by seasonal changes.** | **Control for age and season effects.** | **Grave et al. studied the association between seasonal influenza vaccination and Guillan Barre syndrome. They adjusted for calendar month, as the vaccinations are seasonal by design.**(3)  **In a study of the association between chickenpox and stroke, Thomas et al. adjusted for age in 5-year age bands.**(4) |
| **Events are independently recurrent or rare.** | **MI and stroke are not independent: once an individual has a first event, they are more likely to have a second.** | **Study first events only.** | **Langan et al. began the observation period 12 months into follow-up time to ensure first stroke events had been correctly identified.**(1) |

e-Table 1 Model assumptions and solutions to violations of those assumptions

| **Dataset** | **Description** | **Data Coverage and Representativeness** |
| --- | --- | --- |
| Patient Episode Database for Wales (PEDW) | The PEDW data contains International Classification of Disease Version 10 (ICD-10) coded diagnoses for individuals admitted to any Welsh hospital and Welsh residents treated in English hospitals. This includes all inpatient and day case activity and includes both spell and episode level data. After the patient is discharged, handwritten notes are transcribed at each hospital into ICD-10 codes. | All Welsh hospital admissions, and Welsh residents treated in English hospitals, between 01/04/1995 and 05/02/2024. |
| Welsh Longitudinal General Practice Dataset (WLGP) | The WLGP includes attendance and clinical information for all general practice interactions at included practices. It includes data on demographics, symptoms, diagnoses, prescriptions and referrals. It can be linked to other datasets via anonymised fields for individuals and GPs. An electronic health record for each patient is maintained at the general practice on a clinical information system, with most data entered during the consultation with a clinician. Test results from secondary care are transferred into the system electronically. The majority of clinicians use Read Code terminology, but there are no standard rules for recording in primary care. | 83% of GP practices in Wales. 86% of Welsh population. 01/01/2000 - 01/07/2021. |
| Welsh Results Reporting Service (WRRS) | The WRRS contains laboratory results from across all health boards in Wales. The service allows clinicians to access results regardless of where they were requested or tested, saving time, test duplication and improving patient safety. | All tests requested from primary and secondary care NHS Wales organisations processed and analysed in NHS Wales laboratories. 01/06/1992 - 29/06/2021. |

e-Table 2 Description of SAIL datasets and the coverage of the Welsh population

| Code | Description |
| --- | --- |
| 1A1.. | Urinary frequency/Frequency of micturition/Micturition frequency/Polyuria |
| 1A12. | Frequency of micturition |
| 1A1Z. | Micturition frequency NOS |
| 1A44. | Urine looks cloudy |
| 1A45. | Blood in urine - haematuria/Blood in urine - symptom/Haematuria - symptom |
| 1A55. | Dysuria |
| 1AG.. | Recurrent urinary tract infections |
| 1AZ6. | Lower urinary tract symptoms |
| 1J4.. | Suspected UTI |
| K0A2. | Recurrent and persistent haematuria |
| K101. | Acute pyelonephritis |
| K101z | Acute pyelonephritis NOS |
| K10y0 | Pyelonephritis unspecified |
| K15.. | Cystitis |
| K150. | Acute cystitis |
| K152. | Other chronic cystitis |
| K152y | Chronic cystitis unspecified |
| K152z | Other chronic cystitis NOS |
| K155. | Recurrent cystitis |
| K15yz | Other cystitis NOS |
| K15z. | Cystitis NOS |
| K190. | Recurrent urinary tract infection/Urinary tract infection, site not specified |
| K1903 | Recurrent UTI/Recurrent urinary tract infection |
| K1905 | Urinary tract infection |
| K190z | Urinary tract infection, site not specified NOS |
| K1970 | Painless haematuria |
| K1971 | Painful haematuria |
| K1973 | Frank haematuria |
| Kyu51 | [X]Other cystitis |
| L1668 | Urinary tract infection complicating pregnancy |
| R08.. | [D]Urinary system symptoms |
| R081. | [D]Dysuria |
| R081z | [D]Dysuria NOS |
| R084. | [D]Micturition frequency and polyuria |
| R0840 | [D]Frequency of micturition, unspecified |
| R0842 | [D]Nocturia |
| R084z | [D]Frequency of micturition or polyuria NOS |
| R0908 | [D]Suprapubic pain |
| SP07Q | Catheter-associated urinary tract infection/CAUTI - catheter-associated urinary tract infection |

e-Table 3 Read codes for urinary tract infection to determine risk periods

| Code | Description |
| --- | --- |
| e31B. | *AMIX 250mg/5mL suspension |
| e319. | *AMOXIL SF 125mg/5mL syrup |
| e31a. | *AMOXIL SF 250mg/5mL syrup |
| e31d. | *AMOXIL SF 3g sachets |
| e31R. | *AMOXYMED 250mg capsules |
| e31i. | *AUGMENTIN 375mg disp tablets |
| e69e. | *CEPOREX 250mg/5mL suspension |
| eg68. | *CIPROXIN 100mg tablets |
| e31v. | *CO-AMOXICLAV 125mg/5mL susp |
| e31u. | *CO-AMOXICLAV 375mg disp tabs |
| egA3. | *FOSFOMYCIN 3g/sachet granules |
| eg17. | *MACRODANTIN 100mg capsules |
| eg16. | *MACRODANTIN 50mg capsules |
| eccb. | *MONOTRIM 50mg/5mL s/f susp |
| egA1. | *MONURIL 3g/sach granules |
| ecc3. | *TRIMETHOPRIM 300mg tablets |
| e3z5. | AMIX 250mg capsules |
| e3z6. | AMIX 500mg capsules |
| e3zo. | AMOXICILLIN 125mg/1.25mL susp |
| e3zk. | AMOXICILLIN 125mg/5mL s/f susp |
| e3zm. | AMOXICILLIN 125mg/5mL syrup |
| e311. | AMOXICILLIN 250mg capsules |
| e3zu. | AMOXICILLIN 250mg/5mL s/f susp |
| e3zn. | AMOXICILLIN 250mg/5mL syrup |
| e312. | AMOXICILLIN 500mg capsules |
| e3zq. | AMOXICILLIN powder 3g/sachet |
| e31b. | AMOXIL 125mg/1.25mL paed susp |
| e315. | AMOXIL 250mg capsules |
| e316. | AMOXIL 500mg capsules |
| e3zo. | AMOXYCILLIN 125mg/1.25mL susp |
| e3zk. | AMOXYCILLIN 125mg/5mL s/f susp |
| e3zm. | AMOXYCILLIN 125mg/5mL syrup |
| e311. | AMOXYCILLIN 250mg capsules |
| e3zu. | AMOXYCILLIN 250mg/5mL s/f susp |
| e3zn. | AMOXYCILLIN 250mg/5mL syrup |
| e312. | AMOXYCILLIN 500mg capsules |
| e3zq. | AMOXYCILLIN powder 3g/sachet |
| e31k. | AUGMENTIN 125/31 in 5mL susp |
| e31P. | AUGMENTIN 250/62 in 5mL susp |
| e31h. | AUGMENTIN 375mg tablets |
| e31T. | AUGMENTIN 625mg tablets |
| e31Y. | AUGMENTIN-DUO 400/57in5mL susp |
| e61C. | CEFACLOR 125mg/5mL s/f susp |
| e615. | CEFACLOR 125mg/5mL suspension |
| e614. | CEFACLOR 250mg capsules |
| e61D. | CEFACLOR 250mg/5mL s/f susp |
| e616. | CEFACLOR 250mg/5mL suspension |
| e61a. | CFACLOR 375mg m/r tablets |
| e618. | CEFACLOR 500mg capsules |
| e31B. | *AMIX 250mg/5mL suspension |
| e319. | *AMOXIL SF 125mg/5mL syrup |
| e31a. | *AMOXIL SF 250mg/5mL syrup |
| e31d. | *AMOXIL SF 3g sachets |
| e31R. | *AMOXYMED 250mg capsules |
| e31i. | *AUGMENTIN 375mg disp tablets |
| e69e. | *CEPOREX 250mg/5mL suspension |
| eg68. | *CIPROXIN 100mg tablets |
| e31v. | *CO-AMOXICLAV 125mg/5mL susp |
| e31u. | *CO-AMOXICLAV 375mg disp tabs |
| egA3. | *FOSFOMYCIN 3g/sachet granules |
| eg17. | *MACRODANTIN 100mg capsules |
| eg16. | *MACRODANTIN 50mg capsules |
| eccb. | *MONOTRIM 50mg/5mL s/f susp |
| egA1. | *MONURIL 3g/sach granules |
| ecc3. | *TRIMETHOPRIM 300mg tablets |
| e3z5. | AMIX 250mg capsules |
| e3z6. | AMIX 500mg capsules |
| e3zo. | AMOXICILLIN 125mg/1.25mL susp |
| e3zk. | AMOXICILLIN 125mg/5mL s/f susp |
| e3zm. | AMOXICILLIN 125mg/5mL syrup |
| e311. | AMOXICILLIN 250mg capsules |
| e3zu. | AMOXICILLIN 250mg/5mL s/f susp |
| e3zn. | AMOXICILLIN 250mg/5mL syrup |
| e312. | AMOXICILLIN 500mg capsules |
| e3zq. | AMOXICILLIN powder 3g/sachet |
| e31b. | AMOXIL 125mg/1.25mL paed susp |
| e315. | AMOXIL 250mg capsules |
| e316. | AMOXIL 500mg capsules |
| e3zo. | AMOXYCILLIN 125mg/1.25mL susp |
| e3zk. | AMOXYCILLIN 125mg/5mL s/f susp |
| e3zm. | AMOXYCILLIN 125mg/5mL syrup |
| e311. | AMOXYCILLIN 250mg capsules |
| e3zu. | AMOXYCILLIN 250mg/5mL s/f susp |
| e3zn. | AMOXYCILLIN 250mg/5mL syrup |
| e312. | AMOXYCILLIN 500mg capsules |
| e3zq. | AMOXYCILLIN powder 3g/sachet |
| e31k. | AUGMENTIN 125/31 in 5mL susp |
| e31P. | AUGMENTIN 250/62 in 5mL susp |
| e31h. | AUGMENTIN 375mg tablets |
| e31T. | AUGMENTIN 625mg tablets |
| e31Y. | AUGMENTIN-DUO 400/57in5mL susp |
| e61C. | CEFACLOR 125mg/5mL s/f susp |
| e615. | CEFACLOR 125mg/5mL suspension |
| e614. | CEFACLOR 250mg capsules |
| e61D. | CEFACLOR 250mg/5mL s/f susp |
| e616. | CEFACLOR 250mg/5mL suspension |
| e61a. | CEFACLOR 375mg m/r tablets |
| e618. | CEFACLOR 500mg capsules |
| e69.. | CEFALEXIN |
| e695. | CEFALEXIN 125mg/5mL mixture |
| e69v. | CEFALEXIN 125mg/5mL syrup |
| e691. | CEFALEXIN 250mg capsules |
| e693. | CEFALEXIN 250mg tablets |
| e696. | CEFALEXIN 250mg/5mL mixture |
| e69w. | CEFALEXIN 250mg/5mL syrup |
| e692. | CEFALEXIN 500mg capsules |
| e694. | CEFALEXIN 500mg tablets |
| e697. | CEFALEXIN 500mg/5mL syrup |
| e69.. | CEPHALEXIN |
| e695. | CEPHALEXIN 125mg/5mL mixture |
| e69v. | CEPHALEXIN 125mg/5mL syrup |
| e691. | CEPHALEXIN 250mg capsules |
| e693. | CEPHALEXIN 250mg tablets |
| e696. | CEPHALEXIN 250mg/5mL mixture |
| e69w. | CEPHALEXIN 250mg/5mL syrup |
| e692. | CEPHALEXIN 500mg capsules |
| e694. | CEPHALEXIN 500mg tablets |
| e697. | CEPHALEXIN 500mg/5mL syrup |
| e69f. | CEPOREX 125mg/5mL syrup |
| e698. | CEPOREX 250mg capsules |
| e69a. | CEPOREX 250mg tablets |
| e69g. | CEPOREX 250mg/5mL syrup |
| e699. | CEPOREX 500mg capsules |
| e69b. | CEPOREX 500mg tablets |
| e69h. | CEPOREX 500mg/5mL syrup |
| eg6.. | CIPROFLOXACIN |
| eg67. | CIPROFLOXACIN 100mg tablets |
| eg6x. | CIPROFLOXACIN 250mg tablets |
| eg6w. | CIPROFLOXACIN 500mg tablets |
| eg69. | CIPROFLOXACIN 5g/100mL susp |
| eg6v. | CIPROFLOXACIN 750mg tablets |
| eg61. | CIPROXIN 250mg tablets |
| eg64. | CIPROXIN 500mg tablets |
| eg6A. | CIPROXIN 5g/100mL suspension |
| eg65. | CIPROXIN 750mg tablets |
| e31Q. | CO-AMOXICLAV 125/31mg/5mL susp |
| e31z. | CO-AMOXICLAV 250/62in5mL susp |
| e31t. | CO-AMOXICLAV 375mg tablets |
| e31X. | CO-AMOXICLAV 400/57mg susp |
| e31U. | CO-AMOXICLAV 625mg tablets |
| e612. | DISTACLOR 125mg/5mL suspension |
| e613. | DISTACLOR 250mg/5mL suspension |
| e617. | DISTACLOR 500mg capsules |
| e619. | DISTACLOR MR 375mg m/r tablets |
| ebI.. | FOSFOMYCIN |
| eg14. | FURADANTIN 100mg tablets |
| eg13. | FURADANTIN 50mg tablets |
| eg1C. | GENFURA 100mg tablets |
| eg1B. | GENFURA 50mg tablets |
| e69m. | KEFLEX 125mg/5mL suspension |
| e69i. | KEFLEX 250mg capsules |
| e69k. | KEFLEX 250mg tablets |
| e69n. | KEFLEX 250mg/5mL suspension |
| e69j. | KEFLEX 500mg capsules |
| e69l. | KEFLEX 500mg tablets |
| eg1A. | MACROBID 100mg m/r capsules |
| eg17. | MACRODANTIN 100mg capsules |
| eg16. | MACRODANTIN 50mg capsules |
| eg1x. | NITROFURANT 25mg/5mL s/f susp |
| eg1.. | NITROFURANTOIN |
| eg1z. | NITROFURANTOIN 100mg capsules |
| eg1w. | NITROFURANTOIN 100mg m/r caps |
| eg12. | NITROFURANTOIN 100mg tablets |
| eg1y. | NITROFURANTOIN 50mg capsules |
| eg11. | NITROFURANTOIN 50mg tablets |
| e52w. | PIVMECILLINAM HCL 200mg tabs |
| e521. | SELEXID 200mg tablets |
| ecc.. | TRIMETHOPRIM |
| ecc1. | TRIMETHOPRIM 100mg tablets |
| ecc2. | TRIMETHOPRIM 200mg tablets |
| ecc4. | TRIMETHOPRIM 50mg/5mL s/f susp |

e-Table 4 Read codes for antibiotics to determine risk periods

| Code | Description | Condition |
| --- | --- | --- |
| I210 | Acute transmural myocardial infarction of anterior wall | Myocardial Infarction |
| I211 | Acute transmural myocardial infarction of inferior wall | Myocardial Infarction |
| I212 | Acute transmural myocardial infarction of other sites | Myocardial Infarction |
| I213 | Acute transmural myocardial infarction of unspecified site | Myocardial Infarction |
| I214 | Acute subendocardial myocardial infarction | Myocardial Infarction |
| I219 | Acute myocardial infarction unspecified | Myocardial Infarction |
| I220 | Subsequent myocardial infarction of anterior wall | Myocardial Infarction |
| I221 | Subsequent myocardial infarction of inferior wall | Myocardial Infarction |
| I228 | Subsequent myocardial infarction of other sites | Myocardial Infarction |
| I229 | Subsequent myocardial infarction of unspecified site | Myocardial Infarction |
|  |  |  |
| I600 | Subarachnoid haemorrhage from carotid siphon and bifurcation | STROKE |
| I601 | Subarachnoid haemorrhage from middle cerebral artery | STROKE |
| I602 | Subarachnoid haemorrhage from anterior communicating artery | STROKE |
| I603 | Subarachnoid haemorrhage from posterior communicating artery | STROKE |
| I604 | Subarachnoid haemorrhage from basilar artery | STROKE |
| I605 | Subarachnoid haemorrhage from vertebral artery | STROKE |
| I606 | Subarachnoid haemorrhage from other intracranial arteries | STROKE |
| I607 | Subarachnoid haemorrhage from intracranial artery unspec | STROKE |
| I608 | Other subarachnoid haemorrhage | STROKE |
| I609 | Subarachnoid haemorrhage unspecified | STROKE |
| I610 | Intracerebral haemorrhage in hemisphere subcortical | STROKE |
| I611 | Intracerebral haemorrhage in hemisphere cortical | STROKE |
| I612 | Intracerebral haemorrhage in hemisphere unspecified | STROKE |
| I613 | Intracerebral haemorrhage in brain stem | STROKE |
| I614 | Intracerebral haemorrhage in cerebellum | STROKE |
| I615 | Intracerebral haemorrhage intraventricular | STROKE |
| I616 | Intracerebral haemorrhage multiple localized | STROKE |
| I618 | Other intracerebral haemorrhage | STROKE |
| I619 | Intracerebral haemorrhage unspecified | STROKE |
| I629 | Intracranial haemorrhage (nontraumatic)unspecified | STROKE |
| I630 | Cerebral infarct due to thrombosis of precerebral arteries | STROKE |
| I631 | Cerebral infarction due to embolism of precerebral arteries | STROKE |
| I632 | Cereb infarct due unsp occlusion or stenos precerebrl arts | STROKE |
| I633 | Cerebral infarction due to thrombosis of cerebral arteries | STROKE |
| I634 | Cerebral infarction due to embolism of cerebral arteries | STROKE |
| I635 | Cerebrl infarct due unspec occlusion or stenos cerebrl arts | STROKE |
| I636 | Cerebral infarction due to cerebral venous thrombosis, nonpyogenic | STROKE |
| I638 | Other cerebral infarction | STROKE |
| I639 | Cerebral infarction unspecified | STROKE |
| I64X | Stroke not specified as haemorrhage or infarction | STROKE |

e-Table 5 ICD-10 codes for acute myocardial infarction and stroke to identify potential cases

|  | Number of Individuals | | Total Exposures to Urinary Tract Infection | |
| --- | --- | --- | --- | --- |
|  | MI | Stroke | MI | Stroke |
| Cases | 51,660 | 58,150 | - | - |
| Primary: microbiologically confirmed | 2,320 (4∙5%) | 2,840 (4∙9%) | 3,900 | 4,600 |
| Secondary 1: mixed bacterial growth on culture | 1,110 (2∙1%) | 1,620 (2∙8%) | 1,490 | 2,140 |
| Secondary 2: clinically diagnosed and treated | 11,020 (21∙3%) | 14,260 (24∙5%) | 32,900 | 42,430 |
| Secondary 3: no growth on culture | 1,540 (3∙0%) | 1,640 (2∙8%) | 2,010 | 2,070 |

e-Table 6 Number of individuals and exposures to urinary tract infection included in each analysis. MI= Myocardial Infarction.

| **Number of_UTIs** | **MI*** | **Stroke*** |
| --- | --- | --- |
| 1 | 1560 | 1990 |
| 2 | 410 | 460 |
| 3 | 170 | 190 |
| 4 | 70 | 90 |
| 5 | 40 | 40 |
| 6 | 20 | 30 |
| 7 | <5 | 10 |
| 8 | 10 | 10 |
| 9 | <5 | 10 |
| 10+ | 20 | 10 |

e-Table 7 Number of UTIs per individual in the primary analysis. MI=Myocardial Infarction.

*Rounded to the nearest 10

|  | MI* | Stroke* |
| --- | --- | --- |
| Amoxicillin (n, %) | 280 (7%) | 295 (6%) |
| Cefalexin (n, %) | 340 (9%) | 395 (9%) |
| Ciprofloxacin (n, %) | 180 (5%) | 185 (4%) |
| Nitrofurantoin (n, %) | 1290 (33%) | 1610 (35%) |
| Trimethoprim (n, %) | 1665 (43%) | 1955 (42%) |
| Other (n, %) | 145 (4%) | 155 (3%) |

e-Table 8 Number of urinary tract infections prescribed each antibiotic in the primary analysis. MI= Myocardial Infarction.

*rounded to the nearest 5.

**Secondary Analysis Results**

|  | **MI** | | | | **Stroke** | | | |
| --- | --- | --- | --- | --- | --- | --- | --- | --- |
| **Time period** | **No Events*** | **Total Obs Time (days)** | **Crude IRR (95% CI)** | **Adjusted IRR (95% CI)** | **No Events*** | **Total Obs Time (days)** | **Crude IRR (95% CI)** | **Adjusted IRR (95% CI)** |
| **Baseline** | 1060 | 3,733,366 | 1 | 1 | 1510 | 5,358,276 | 1 | 1 |
| **Pre-Risk** | ≤5 | 10,500^+^ | - | 0∙50 (0∙13 - 2∙02) | ≤5 | 15,000^+^ | - | 0∙16 (0∙02 - 1∙17) |
| **1-7 days** | ≤5 | 10400^+^ | - | 1.26 (0.52 - 3.05) | 20 | 14,925 | 4.20 (2.67 - 6.63) | 3.14 (1.99 - 4.95) |
| **8-14 days** | 10 | 10,189 | 2∙59 (1∙29 - 5∙19) | 2∙07 (1∙03 - 4∙15) | 10 | 14,684 | 2∙28 (1∙22 - 4∙26) | 1∙70 (0∙91 - 3∙17) |
| **15-28 days** | 10 | 20,010 | 1∙32 (0∙66 - 2∙65) | 1∙05 (0∙52 - 2∙12) | 20 | 28,752 | 2∙10 (1∙32 - 3∙35) | 1∙56 (0∙98 - 2∙49) |
| **29-90 days** | 30 | 83,437 | 1∙15 (0∙80 - 1∙67) | 0∙92 (0∙63 - 1∙34) | 60 | 120,165 | 1∙70 (1∙31 - 2∙21) | 1∙26 (0∙97 - 1∙64) |

e-Table 9 Crude, and age-, season- and year-adjusted incidence rate ratio (IRR) for myocardial infarction and stroke in periods after urinary tract infection compared with baseline time for secondary analysis 1: mixed bacterial growth on culture. MI= Myocardial Infarction. IRR= Incidence Rate Ratio. CI= Confidence Interval.

*rounded to the nearest 10.

^+^ rounded to the nearest 100.

- Excluded for disclosure reasons

|  | **MI** | | | | **Stroke** | | | |
| --- | --- | --- | --- | --- | --- | --- | --- | --- |
| **Time period** | **No Events*** | **Total Obs Time (days)** | **Crude IRR (95% CI)** | **Adjusted IRR (95% CI)** | **No Events*** | **Total Obs Time (days)** | **Crude IRR (95% CI)** | **Adjusted IRR (95% CI)** |
| **Baseline** | 10090 | 34,221,259 | 1 | 1 | 12820 | 41,785,087 | 1 | 1 |
| **Pre-Risk** | 20 | 209,529 | 0∙30 (0∙19 - 0∙46) | 0∙28 (0∙18 - 0∙43) | 20 | 269,823 | 0∙21 (0∙14 - 0∙33) | 0∙19 (0∙12 - 0∙29) |
| **1-7 days** | 130 | 199,980 | 1.95 (1.64 - 2.33) | 1.83 (1.54 - 2.18) | 250 | 258,930 | 2.74 (2.41 - 3.11) | 2.43 (2.14 - 2.76) |
| **8-14 days** | 90 | 192,127 | 1∙43 (1∙16 - 1∙76) | 1∙34 (1∙09 - 1∙65) | 160 | 249,908 | 1∙85 (1∙58 - 2∙16) | 1∙64 (1∙40 - 1∙92) |
| **15-28 days** | 170 | 365,683 | 1∙38 (1∙18 - 1∙61) | 1∙30 (1∙11 - 1∙51) | 250 | 475,620 | 1∙51 (1∙33 - 1∙71) | 1∙34 (1∙18 - 1∙52) |
| **29-90 days** | 520 | 1,433,513 | 1∙10 (1∙00 - 1∙20) | 1∙03 (0∙94 - 1∙13) | 760 | 1,854,564 | 1∙19 (1∙11 - 1∙29) | 1∙06 (0∙98 - 1∙14) |

e-Table 10 Crude, and age-, season- and year-adjusted incidence rate ratio (IRR) for myocardial infarction and stroke in periods after urinary tract infection compared with baseline time for secondary analysis 2: clinically diagnosed and treated urinary tract infection. MI= Myocardial Infarction. IRR= Incidence Rate Ratio. CI=Confidence Interval.

*rounded to the nearest 10.

|  | **MI** | | | | **Stroke** | | | |
| --- | --- | --- | --- | --- | --- | --- | --- | --- |
| **Time period** | **No Events*** | **Total Obs Time (days)** | **Crude IRR (95% CI)** | **Adjusted IRR (95% CI)** | **No Events*** | **Total Obs Time (days)** | **Crude IRR (95% CI)** | **Adjusted IRR (95% CI)** |
| **Baseline** | 1470 | 5,407,559 | 1 | 1 | 1530 | 5,595,427 | 1 | 1 |
| **Pre-Risk** | ≤5 | 14,100^+^ | - | 0∙22 (0∙03 - 1∙53) | ≤5 | - | - | - |
| **1-7 days** | 20 | 14,035 | 4.24 (2.62 - 6.84) | 3.69 (2.28 - 5.96) | 20 | 14,455 | 4.05 (2.51 - 6.53) | 3.20 (1.98 - 5.17) |
| **8-14 days** | ≤5 | 13,900^+^ | - | 0∙88 (0∙33 - 2∙35) | 20 | 14,249 | 5∙08 (3∙30 - 7∙83) | 4∙02 (2∙61 - 6∙20) |
| **15-28 days** | 10 | 27,398 | 1∙15 (0∙60 - 2∙23) | 1∙00 (0∙52 - 1∙94) | 20 | 28,097 | 2∙09 (1∙30 - 3∙38) | 1∙65 (1∙02 - 2∙67) |
| **29-90 days** | 40 | 116,223 | 1∙27 (0∙94 - 1∙74) | 1∙10 (0∙81 - 1∙50) | 60 | 119,615 | 1∙81 (1∙40 - 2∙34) | 1∙41 (1∙09 - 1∙83) |

e-Table 11 Crude, and age-, season- and year-adjusted incidence rate ratio (IRR) for myocardial infarction and stroke in periods after urinary tract infection compared with baseline time for secondary analysis 3: no growth on culture. MI= Myocardial Infarction. IRR= Incidence Rate Ratio. CI= Confidence Interval.

*rounded to the nearest 10.

^+^ rounded to the nearest 100.

- excluded for disclosure reasons

**Subgroup and Sensitivity Analysis Results**

|  | **MI** | | **Stroke** | |
| --- | --- | --- | --- | --- |
| **Sensitivity analyses (0-7 days)** | **No Events*** | **Adjusted IRR (95% CI)** | **No Events*** | **Adjusted IRR (95% CI)** |
| Wider definition of MI/stroke | 2300 | 2.52 (1.62 - 3.93) | 2850 | 2.20 (1.49 - 3.25) |
| Wider definition of UTI | 3190 | 2.40 (1.70 - 3.40) | 3900 | 2.11 (1.53 - 2.91) |
| First ever MI/stroke | 2120 | 2.61 (1.71 - 3.98) | 2730 | 2.34 (1.60 - 3.42) |
| 14-day pre-risk period | 2310 | 2.54 (1.68 - 3.85) | 2850 | 2.37 (1.63 - 3.45) |
| Exclude individuals who died within 30 days of event | 2190 | 2.25 (1.43 - 3.55) | 2570 | 2.24 (1.48 - 3.39) |
| Nitrofurantoin only | 1170 | 2.39 (1.24 - 4.63) | 1470 | 2.51 (1.45 - 4.35) |
| Trimethoprim only | 1390 | 3.01 (1.74 - 5.22) | 1640 | 2.53 (1.49 - 4.29) |
| Ischaemic stroke only | - | - | 2000 | 2.27 (1.46 - 3.55) |
| COVID-19: end observation period after 2019 | 2050 | 2.63 (1.74 - 3.98) | 2440 | 2.22 (1.49 - 3.30) |
| COVID-19: Pre-2020 vs 2020 interaction |  |  |  |  |
| Pre-2020 | 2120 | 2.71 (1.79 - 4.10) | 2510 | 2.35 (1.58 - 3.50) |
| 2020 | 180 | - | 320 | 2.22 (0.71 - 6.98) |
| **Sub-groups** |  |  |  |  |
| Organism |  |  |  |  |
| E∙coli | 1630 | 2.55 (1.37 - 4.76) | 1960 | 3.06 (1.87 – 5.00) |
| Other | 680 | 3.54 (1.58 - 7.91) | 890 | 2.34 (1.05 - 5.23) |
| Diabetes status |  |  |  |  |
| Yes | 700 | 1.41 (0.52 - 3.77) | 840 | 2.39 (1.23 - 4.62) |
| No | 1600 | 2.97 (1.88 - 4.69) | 2000 | 2.32 (1.47 - 3.65) |
| Gender  Male  Female | 900  1420 | 2.49 (1.24 – 5.00)  2.50 (1.50 - 4.17) | 890  1950 | 2.53 (1.31 - 4.90)  2.26 (1.43 - 3.56) |

e-Table 12 Age-, season- and year-adjusted incidence rate ratio (IRR) for myocardial infarction and stroke in the first seven days after urinary tract infection compared with baseline time for subgroup and sensitivity analyses. Subsequent risk periods are excluded for brevity. MI= Myocardial Infarction. IRR= Incidence Rate Ratio. CI= Confidence Interval

*Total number of events included in analysis, rounded to the nearest 10.

**References**

1. Gibson JE, Hubbard RB, Smith CJP, Tata LJ, Britton JR, Fogarty AW. Use of Self-controlled Analytical Techniques to Assess the Association Between Use of Prescription Medications and the Risk of Motor Vehicle Crashes. Am J Epidemiol. 2009 Jan 6;169(6):761–8.

2. Langan SM, Minassian C, Smeeth L, Thomas SL. Risk of Stroke Following Herpes Zoster: A Self-Controlled Case-Series Study. Clinical Infectious Diseases. 2014 Jun 1;58(11):1497–503.

3. Grave C, Boucheron P, Rudant J, Mikaeloff Y, Tubert-Bitter P, Escolano S, et al. Seasonal influenza vaccine and Guillain-Barré syndrome. Neurology. 2020 May 19;94(20).

4. Thomas SL, Minassian C, Ganesan V, Langan SM, Smeeth L. Chickenpox and Risk of Stroke: A Self-controlled Case Series Analysis. Clinical Infectious Diseases. 2014 Jan 1;58(1):61–8.
