# Supplementary material for: Risk of myocardial infarction and stroke following microbiologically confirmed urinary tract infection: a self-controlled case series study using linked electronic health data
Source: BMJ Open. 2025 Jun 30;15(6):e097754. doi: 10.1136/bmjopen-2024-097754 (PMC12211855; doi:10.1136/bmjopen-2024-097754)
Supplement: Supplementary file 2 [file bmjopen-2024-097754.reviewer_comments.pdf]

PEER REVIEW HISTORY

BMJ Open publishes all reviews undertaken for accepted manuscripts. Reviewers are asked to complete a checklist review form and are provided with free text boxes to elaborate on their assessment. These free text comments are reproduced below.

This paper was submitted to another journal from BMJ but declined for publication following peer review. The authors addressed the reviewers' comments and submitted the revised paper to BMJ Open. The paper was subsequently accepted for publication at BMJ Open.

ARTICLE DETAILS

Title (Provisional)

Risk of myocardial infarction and stroke following microbiologically confirmed urinary tract infection: a self-controlled case series study using linked electronic health data

Authors

Reeve, Nicola F; Best, Victoria; Cannings-John, Rebecca; Gillespie, David; Hughes, Kathryn; Lugg-Widger, Fiona V; Torabi, Fatemeh; Wootton, Mandy; Akbari, Ashley; Ahmed, Haroon

---

VERSION 1 - REVIEW

**Reviewer**	**1**
**Name**	**Fors Connolly, Anne-Marie**
**Affiliation**	**Umeå Universitet Medicinska fakulteten, Dept. of Clinical Microbiology**
**Date**	**28-Jun-2024**
**COI**	**None**

Risk of myocardial infarction and stroke following microbiologically confirmed urinary tract infection: a self-controlled case series study using linked electronic health data

This is a study of acute cardiovascular events following microbiologically confirmed urinary tract infection. This is an important study, however, there are some issues that need to be addressed.

Major comments:
Please do not include day 0 in the first risk period, since this risk introducing bias. Please refer to the paper by Fonseca-Rodriguez et al, that identifies the risk of including day 0 in the SCCS method (https://onlinelibrary.wiley.com/doi/abs/10.1002/sim.9179)

The mechanisms of action for stroke differ for ischemic and hemorrhagic stroke. Suggestion to divide these into ischemic and hemorrhagic outcomes.

The adjusted IRRs for MI is significant in the first week (including day 0) and 15-28 days. It is not significant during 8-14 days. Therefore, to write that the IRR decreased to baseline after 28 days is not

correct. Please amend this, and please repeat the study to show day 0 separately. This needs to be adjusted both in the results section and the discussion.

The adjusted IRRs for stroke is only significant after in days 0-7 and 29-90 days, and not significant in the time period in between. Therefore, please amend the sentence "The risk of stroke also declined over time but unlike MI, remained raised for at least 90 days following UTI). This is not quite correct. This needs to be adjusted both in the results section and the discussion.

Minor comments:
Summary
- I would suggest that you remove the word "causal" from the objectives, since in order to study the cause between infection and outcome, you would need in vitro or in vivo studies. In these epidemiological studies, you can identify associations.
- Why welsh residents over 30 years of age only and not younger?

Introduction
- Very nice introduction and background to the research field

Methods
- Please provide a summary of the study procedures. The likelihood of a reader reading another document in order to understand the methodology is slim, therefore to increase readability, please include the details in the original manuscript.
- Regarding the SAIL databank, does this include all Welsh citizens? Or a fraction of Welsh citizens? Please specify how many of the Welsh population that could be expected to be in this database.
- Is it possible to go further back than 2010 to make sure that this is the first AMI or stroke that you identify for each individual?

**Reviewer**	**2**
**Name**	**Reviewer 2**
**Affiliation**	
**Date**	**28-Jun-2024**
**COI**	**None**

Comments not published pending reviewer's consent

**Reviewer**	**3**
**Name**	**Reviewer 3**
**Affiliation**	
**Date**	**28-Jun-2024**
**COI**	**None**

Comments not published pending reviewer's consent

VERSION 1 - AUTHOR RESPONSE

Reviewer: 1

Recommendation:

Comments:

Risk of myocardial infarction and stroke following microbiologically confirmed urinary tract infection: a self-controlled case series study using linked electronic health data

This is a study of acute cardiovascular events following microbiologically confirmed urinary tract infection. This is an important study, however, there are some issues that need to be addressed.

Major comments:

Please do not include day 0 in the first risk period, since this risk introducing bias. Please refer to the paper by Fonseca-Rodriguez et al, that identifies the risk of including day 0 in the SCCS method (https://onlinelibrary.wiley.com/doi/abs/10.1002/sim.9179)

All models now include a separate day 0 risk period, followed by a 1-7 days risk period. The results have been updated in the Results section, Table 3, Figures 3 and 4 and Supplementary Material.

The mechanisms of action for stroke differ for ischemic and hemorrhagic stroke. Suggestion to divide these into ischemic and hemorrhagic outcomes.

We acknowledge the potential importance of this difference. We have added a sensitivity analysis restricting the definition of stroke to ischaemic stroke only.See e-table 12.

The adjusted IRRs for MI is significant in the first week (including day 0) and 15-28 days. It is not significant during 8-14 days. Therefore, to write that the IRR decreased to baseline after 28 days is not correct. Please amend this, and please repeat the study to show day 0 separately. This needs to be adjusted both in the results section and the discussion.

Amended in text, and all models are now updated with a separate day 0 risk period.

The adjusted IRRs for stroke is only significant after in days 0-7 and 29-90 days, and not significant in the time period in between. Therefore, please amend the sentence "The risk of stroke also declined over time but unlike MI, remained raised for at least 90 days following UTI). This is not quite correct. This needs to be adjusted both in the results section and the discussion.

Amended in text.

Minor comments:

Summary

- I would suggest that you remove the word "causal" from the objectives, since in order to study the cause between infection and outcome, you would need in vitro or in vivo studies. In these epidemiological studies, you can identify associations.

We have removed all reference to causality.

- Why welsh residents over 30 years of age only and not younger?

As stated in the Study Design and Population section (Page 5, line 23), we chose a lower age bound of 30 years to reduce the chance of including MIs and strokes due to congenital or other non-artherosclerotic causes. A lower age bound of 40 might reduce this chance further but would increase the chance of missing relevant events, especially given the greater burden of cardiovascular disease in Wales compared to the UK as a whole.

Introduction

- Very nice introduction and background to the research field

Methods

- Please provide a summary of the study procedures. The likelihood of a reader reading another document in order to understand the methodology is slim, therefore to increase readability, please include the details in the original manuscript.

All key details of the study procedures are included in this paper. A notable exception was details of the assumptions of the SCCS methodology and how violations would affect our study. This has now been added to the supplementary material in new e-Table 1. Also information on the population included in the SAIL databank – see next comment.

- Regarding the SAIL databank, does this include all Welsh citizens? Or a fraction of Welsh citizens? Please specify how many of the Welsh population that could be expected to be in this database.

A table with details on the SAIL datasets used and their coverage has been added to the Supplementary Material (e-Table 2).

- Is it possible to go further back than 2010 to make sure that this is the first AMI or stroke that you identify for each individual?

This is covered in sensitivity analysis 3 – First-ever MI/stroke, where individuals with MI/stroke before the start of the observation period (2010) were excluded. See methods (Page 7, line 42) and results in Supplementary Material e-Table 10

VERSION 2 - REVIEW

**Reviewer**	**1**
**Name**	**Fors Connolly, Anne-Marie**
**Affiliation**	**Umeå Universitet Medicinska fakulteten, Dept. of Clinical Microbiology**
**Date**	**24-Feb-2025**
**COI**	

No further comments

**Reviewer**	**4**
**Name**	**Dack, Kyle**
**Affiliation**	**University of Bristol, MRC Integrative Epidemiology Unit**
**Date**	**22-Mar-2025**
**COI**	**None**

This paper has already been reviewed, and therefore I do not have many comments to add regarding the methodological or statistical presentation. Most of it is clear and comprehensive already.

I can see there has been discussion regarding the risk of confounding, and my feedback on this is shared below:

Several prior reviewers expressed concern regarding residual or latent confounding. The authors accurately point out that SCCS controls for time-invariant confounding – any factor which does not change over the observation period would not impact the estimates, even if unmeasured.

I agree that in most cases, this should minimize the risk of confounding. The study period is 90 days per individual which in theory could be long enough for time-varying factors to express an influence on CVD. However, the exposure (UTI) is only measured at baseline. So even if there are time-variant factors across the 90 days (such as age), it is not possible for these to impact UTI status which was not measured after day 1. This would also minimize confounding from latent conditions which vary with age and may increase risk of both UTI and CVD.

However, I do have two concerns regarding risk of confounding:

(a) Adjusted covariates (age, season, year of UTI diagnosis) all do have an element of measurement error. Each is aiming to adjust for time, but these are categorical measures of a continuous scale, so there is some loss of precision and this could introduce residual confounding.

I doubt that this would have a major impact – but it should at least be acknowledged as a limitation. Currently the discussion states that the SCCS eliminates residual confounding (strengths and limitations, line 27), which I don't think is accurate. The SCCS eliminates unmeasured time-invariant confounding. Residual confounding is different – it can occur from measurement error in adjusted covariates, which is still possible in this study.

(b) The study is based on EHR data, which can be challenging because measurement procedures, methods, even recording software all change over time, and this can impact levels of missingness or accuracy over time. This study has a 10-year study period. Were there any significant changes in data quality which needed to be addressed in creation of the

datasets or analysis? If there were no major data issues it would still be useful to state this, because future readers may have doubts. The issue is briefly touched on (data sources, lines 36-38), but it would be helpful to have more information about whether it impacted the study or not.

Minor comments:

Line 27: there is an extra fullstop.

References are not consistently positioned – sometimes they are to the left of punctuation (e.g. introduction line 8), sometimes on the right (e.g. introduction line 5).

Missingness: In the STROBE checklist item 14b is marked as NA. I am unsure why – there is missingness reported in Table 2. It would be helpful to have a statement in the results describing the overall level of missingness. Is it only ethnicity where there was missing data? This should be stated because it could impact the interpretation of the main model (whether it is complete-cases only, or includes all possible participants).

Table 3. Although the analysis is at the event-level, it would be helpful to the reader if the table label included the number of participants. This is already in the text, but some readers may overlook this.

VERSION 2 - AUTHOR RESPONSE

Reviewer: 1

Dr. Anne-Marie Fors Connolly, Umeå Universitet Medicinska fakulteten

Comments to the Author:

No further comments

Reviewer: 4

Dr. Kyle Dack, University of Bristol

Comments to the Author:

This paper has already been reviewed, and therefore I do not have many comments to add regarding the methodological or statistical presentation. Most of it is clear and comprehensive already.

I can see there has been discussion regarding the risk of confounding, and my feedback on this is shared below:

Several prior reviewers expressed concern regarding residual or latent confounding. The authors accurately point out that SCCS controls for time-invariant confounding – any factor which does not change over the observation period would not impact the estimates, even if unmeasured.

I agree that in most cases, this should minimize the risk of confounding. The study period is 90 days per individual which in theory could be long enough for time-varying factors to express an

influence on CVD. However, the exposure (UTI) is only measured at baseline. So even if there are time-variant factors across the 90 days (such as age), it is not possible for these to impact UTI status which was not measured after day 1. This would also minimize confounding from latent conditions which vary with age and may increase risk of both UTI and CVD.

However, I do have two concerns regarding risk of confounding:

(a) Adjusted covariates (age, season, year of UTI diagnosis) all do have an element of measurement error. Each is aiming to adjust for time, but these are categorical measures of a continuous scale, so there is some loss of precision and this could introduce residual confounding.

I doubt that this would have a major impact – but it should at least be acknowledged as a limitation. Currently the discussion states that the SCCS eliminates residual confounding (strengths and limitations, line 27), which I don't think is accurate. The SCCS eliminates unmeasured time-invariant confounding. Residual confounding is different – it can occur from measurement error in adjusted covariates, which is still possible in this study.

We have amended the discussion, now line 30 to say 'which meant that unmeasured time-invariant confounding ... were eliminated'.

We have added the sentence 'However, the findings could still be affected by residual confounding, such as from measurement error in adjusted covariates.' To the discussion, line 34.

We have also included 'Residual confounding may still be an issue.' In the new 'Strengths and limitations of the study' section, after the abstract.

(b) The study is based on EHR data, which can be challenging because measurement procedures, methods, even recording software all change over time, and this can impact levels of missingness or accuracy over time. This study has a 10-year study period. Were there any significant changes in data quality which needed to be addressed in creation of the datasets or analysis? If there were no major data issues it would still be useful to state this, because future readers may have doubts. The issue is briefly touched on (data sources, lines 36-38), but it would be helpful to have more information about whether it impacted the study or not.

The key data issue was potentially unrecorded UTIs in the Welsh Results Reporting Service prior to 2015 due to differences in recording practices. This is included in the Strengths and Limitations section of the Discussion, line 40.

Minor comments:

Line 27: there is an extra fullstop.

Amended.

References are not consistently positioned – sometimes they are to the left of punctuation (e.g. introduction line 8), sometimes on the right (e.g. introduction line 5).

Amended.

Missingness: In the STROBE checklist item 14b is marked as NA. I am unsure why – there is missingness reported in Table 2. It would be helpful to have a statement in the results describing the overall level of missingness. Is it only ethnicity where there was missing data? This should be

stated because it could impact the interpretation of the main model (whether it is complete-cases only, or includes all possible participants).

Ethnicity is the only variable with missing data, and doesn't affect the modelling as ethnicity is considered a time-invariant characteristic and therefore adjustment is not needed.

We have added a statement to the Results, page 9 line 3-5 to clarify the missingness and have corrected the STROBE checklist.

Table 3. Although the analysis is at the event-level, it would be helpful to the reader if the table label included the number of participants. This is already in the text, but some readers may overlook this.

As all individuals included in the analysis have experienced an event, the number of events equals the number of participants. However, we agree it is useful for the reader to have this total in the table label, and have added it.
